# Culture versus Policy: More Global Collaboration to Effectively Combat COVID-19

**DOI:** 10.1016/j.xinn.2020.100023

**Published:** 2020-08-01

**Authors:** Jianping Li, Kun Guo, Enrique Herrera Viedma, Heesoek Lee, Jiming Liu, Ning Zhong, Luiz Flavio Autran Monteiro Gomes, Florin Gheorghe Filip, Shu-Cherng Fang, Mujgan Sagir Özdemir, Xiaohui Liu, Guoqing Lu, Yong Shi

**Affiliations:** 1 School of Economics and Management, University of Chinese Academy of Sciences, Beijing 100190, China; 2 Key Laboratory of Big Data Mining and Knowledge Management, Chinese Academy of Sciences, Beijing 100190, China; 3 Research Center on Fictitious Economy & Data Science, Chinese Academy of Sciences, Beijing 100190, China; 4 Department of Computer Science and Artificial Intelligence, E.T.S. de Ingenieria Informatica y de Telecomunicaciones, University of Granada, 18071 Granada, Spain; 5 Department of Information Management, Korea Advanced Institute of Science and Technology, Seoul 207-43 Korea; 6 Department of Computer Science and HKBU-CSD & NIPD Joint Research Laboratory for Intelligent Disease Surveillance and Control, Hong Kong Baptist University, Hong Kong, China; 7 Department of Life Science and Informatics, Maebashi Institute of Technology, Maebashi 371-0816, Japan; 8 Ibmec University Center, Av. Presidente Wilson, 118, Office #1110, 20030-020 Rio de Janeiro, Brazil; 9 The Romanian Academy, Bucharest, 010071, Romania; 10 Industrial and Systems Engineering Department, North Carolina State University, Raleigh, NC 27695, USA; 11 Department of Industrial Engineering, Eskisehir Osmangazi University, 26480 Eskisehir, Turkey; 12 Department of Computer Science, Brunel University London, London, UB8 3PH, UK; 13 Department of Biology and School of Interdisciplinary Informatics, University of Nebraska at Omaha, Omaha, NE 68182, USA; 14 College of Information Science and Technology, University of Nebraska at Omaha, Omaha, NE 68182, USA

## Abstract

The outbreak of COVID-19 seriously challenges every government with regard to capacity and management of public health systems facing the catastrophic emergency. Culture and anti-epidemic policy do not necessarily conflict with each other. All countries and governments should be more tolerant to each other in seeking cultural and political consensus to overcome this historically tragic pandemic together.

## Main Text

The outbreak of COVID-19 has rapidly become a global pandemic. It seriously challenges each government with regard to capacity and management of public health systems facing the catastrophic emergency. Many countries have already achieved early success in controlling the spread of the disease by limiting population movement to near standstill and by effectively redistributing medical resources to meet the demand, while many countries are still in the fight against the ever-fast spread of the disease. This historical pandemic has imperiled the global financial market as well as the world economy to an unprecedented extent, which might trigger plausible geopolitical and social turmoil that most countries must face and get prepared for.

**Figure 1:**
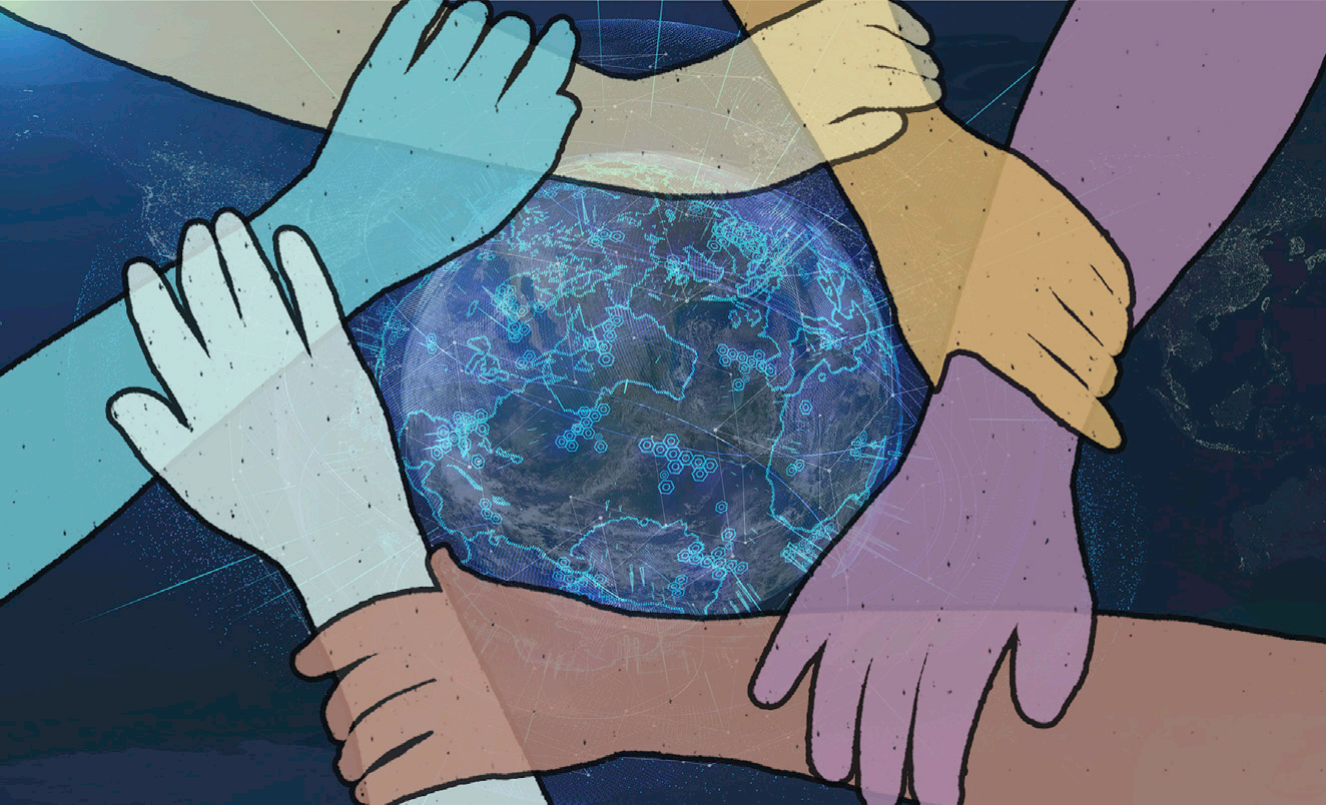
More Global Collaboration to Effectively Combat COVID-19

No single country is an island that can remain immune to the epidemic. What matters most to a country is the actions taken by the government to minimize the negative impacts on the whole society. Global collaboration becomes even more crucial to the mitigation of negative impacts (Figure 1). Good experiences may help to shape effective responses to the outbreak, even though they may have certain limitations from country to country because of differences in cultural and political systems and social or economic development. At the time of writing, the novel coronavirus is still spreading at high speed in America, Russia, Brazil, and African countries. Moreover, many developing countries could experience an even higher risk of having massive outbreaks due to the lack of medical resources, virus detection technology, and accurate epidemic data.

The massive outbreaks across the world have had severe impacts on the global economy and international relations, which are being exacerbated by the measures limiting cross-border movements of people and goods. As a result, every government is making difficult decisions, facing the trade-offs between economic stability and normal social functioning to fight the epidemic. The pessimism generated by watching the ever-increasing daily death toll often reflects on public opinions of “isolating from the danger.” Countries with different social structures, lifestyles, and cultural backgrounds tend to adopt different policies to contain the spread of COVID-19. They should strive to strike a balance between culture and policy.^1^ It is imperative for all countries in the world to come up with an acceptable consensus to embrace the inclusiveness and responsibility of the world community, to put aside national and cultural disparities, and to strengthen international cooperation in order to effectively combat this once-in-a-century pandemic.^2^ Otherwise, we would be fighting a whack-a-mole war in which an individual country's manpower, medical resources, and economic stimulus will become exhausted in the struggle to drive down the impacts of the epidemic.

First, cross-cultural communication and collaboration, especially among scientific and technological communities, should be strengthened in the face of the epidemic. Multilateral collaboration around the world can play an important role in the sharing of experiences, accelerating tracing of the novel coronavirus, vaccine development, and global deployment of epidemic prevention materials. In particular, tracing the new novel coronavirus is the responsibility of the scientific and technological community all over the world, and cooperation and information sharing must be strengthened to accelerate the tackling of this scientific problem.

Second, all countries and governments in the world should strengthen data and information sharing systems to effectively track and trace coronavirus research and jointly develop low-cost testing kits, vaccines, and effective therapeutics under the initiatives of international organizations such as the WHO. Furthermore, the resulting intellectual property rights should be shared by all countries.

Third, with its own cultural and societal considerations, every country or government should seriously adopt the early experiences learned in other countries, including the aspects of “timely and reasonable control of local population flow,” “vast and quick virus tests for the general public,” “extensive use of communication platforms and big data technology to trace the confirmed, quarantined, and contact cases,”^3^ and the coordinated allocation of nationwide medical resources using modern information management platforms to avoid potential saturation of medical capacities.

Fourth, every country and government should take this unique opportunity to evaluate its current public health system, to establish community-based and grid-based “early warning and prevention systems” for public health, and to utilize the latest big data, artificial intelligence, blockchain and advanced computing technologies for sound policy making in preparation for future pandemics.

Fifth, although the WHO and medical societies timely discourage the use of the phrase “social distancing” and recommend the phrase “physical distancing,” just being physically apart without the sense of communal cohesion may aggravates our mental health and well-being issues. All governments should develop novel strategies to promote virtual communities. This should also be accompanied by adequate advocacy and education for the public. The current coronavirus may provide better opportunities to prepare plans for governments to inform society in advance.

Finally, coronavirus shutdowns have yielded unintentional climate and environmental benefits, such as clearer air and cleaner water. Carbon emissions have dropped significantly. However, these benefits may be lost or weakened if governmental policies do not support them when everything gets back to normal.^4^ Therefore, countries should challenge the systems that may possibly destroy the environment, keeping clean environment, air, and water, which are essential to life on the planet.

Culture and anti-epidemic policy do not necessarily conflict with each other. Different societies with various cultural backgrounds will adopt different policies to fight for their best interests in this pandemic. Yet, a consensus of combating COVID-19 collectively and inclusively by all countries and governments is supported by most scientists and economists in the world. The whole world is one community with a shared destiny, as the epidemic may befall anytime, anywhere again, as long as the coronavirus does not disappear. In this context, all countries and governments should be more tolerant of each other in seeking cultural and political consensus that will lead to conquer this historically tragic pandemic together.
